# Determinants of sustained physician engagement in obstetric QI: a TICD-guided qualitative study

**DOI:** 10.1186/s43058-026-00898-y

**Published:** 2026-03-14

**Authors:** Saanie Sulley, Emily Kobernik, Robert J. Sokol

**Affiliations:** 1 National Healthy Start Association, 1325 G Street, N.W. Suite 500, Washington, DC 20005 USA; 2 Department of Learning Health Sciences, Michigan Medicine, Ann Arbor, MI USA; 3 Department of Obstetrics and Gynecology and Department of Physiology, Wayne State University School of Medicine, Detroit, MI USA; 4 Department of Epidemiology and Biostatistics, Michigan State University College of Human Medicine, East Lansing, MI USA

**Keywords:** Quality Improvement, Obstetric Care, Physician Engagement, TICD Framework, Multidisciplinary Collaboration, Leadership, Healthcare Disparities

## Abstract

**Background:**

Maternal health disparities persist despite advances in obstetric care and disproportionately affect historically marginalized populations. Quality Improvement (QI) initiatives, including those led by national and state perinatal quality collaboratives, aim to address these disparities; however, sustaining physician engagement in such initiatives remains challenging. This study applies the Tailored Implementation for Chronic Diseases (TICD) framework to identify key determinants influencing physician participation in QI initiatives within obstetric care.

**Methods:**

A qualitative study was conducted using semi-structured interviews with 24 obstetricians and gynecologists actively engaged in maternal health QI programs across diverse healthcare settings. Participants were selected based on their involvement in QI initiatives. Data were collected between November 2021 and August 2022. Thematic analysis, guided by the TICD framework, categorized facilitators and barriers to engagement, focusing on leadership support, multidisciplinary collaboration, resource availability, and physician motivation. All participants were already engaged in QI; therefore, the study examines determinants of sustained engagement among participating physicians rather than initial participation.

**Results:**

Participants described participatory leadership, interdisciplinary collaboration, and patient-centered motivation as key drivers of sustained QI engagement. Supportive organizational culture and clear role expectations facilitated collaboration and resource allocation. While career advancement and financial incentives were relevant, commitment to improving maternal outcomes was the predominant motivator. Reported barriers included limited time, administrative burden, misalignment between QI priorities and clinical workflows, and insufficient training in QI methods. Physicians emphasized the value of longitudinal QI education and flexible, evidence-based guidelines adaptable to local context and patient populations.

**Conclusions:**

Findings suggest that sustaining physician involvement in QI may be facilitated by participatory leadership, institutional investment (including protected time), and alignment with physician values and patient-centered priorities. Balancing standardization with adaptability and embedding longitudinal QI education were viewed as important for reducing maternal health disparities and improving implementation.

**Supplementary Information:**

The online version contains supplementary material available at 10.1186/s43058-026-00898-y.

Contributions to the literature

- Clarifies determinants of sustained physician engagement in obstetric QI using the TICD framework, based on interviews with physicians already participating in PQC/AIM initiatives.
- Maps facilitators and barriers to standard TICD constructs and flags inductive, OB-specific themes (e.g., L&D workflow constraints, medico-legal context, equity motivations).
- Specifies three leader-actionable strategies (embed QI in roles/time, provide longitudinal QI training, use participatory leadership) linked to implementation outcomes (acceptability, adoption, fidelity, sustainability).

## Introduction

Quality improvement (QI) is central to improving maternal outcomes and addressing inequities in obstetric care. Persistent disparities particularly the markedly higher pregnancy-related mortality among Black women underscore the need to sustain effective QI approaches in maternity care [1, 2]. Perinatal quality collaboratives, regional and national maternal safety initiatives have demonstrated improvments in maternal outcomes [3–5]; however, variability in implementation and sustainability remains a major challenge [6, 7]. Physicians are essential to QI success, yet maintaining engagement is difficult given competing clinical demands, administrative burden, and variable leadership support.

Within implementation and quality improvement literature, clinician and stakeholder engagement is widely recognized as a critical determinants of successful implementation and sustainability, though it is variably defined and operationalized. Engagement has been conceptualized as a multidimentional construst encompassing clinician’s cognitive, behavioral and relational investment in improvement activities, including participation in decision-making, adaptation of interventions, and ongoing use of perfeomance data. Prior studies link clinician engagement to implementation outcome such as adoption, fidelity, and sustainability of evidence-based pacticies. However, engagement is often operationalized as initial buy-in or early participation, with comparatively less attention to the factors that sustain involvement over time. Conceptual frameworks of stakeholder engagement emphasize ongoing interaction, shared ownership, and alignment with professional values, suggesting that engagement is dynamic rather than static. Despite its recognized importance, sustained clinician engagement remains under-theorized in implementation research, particularly within high-acuity clinical context such as obstetrics care [8–11].

Building on these conceptualizations, in this study, physician engagement refers to sustained, active participation in QI activities over time. This includes attending or leading QI meetings and hurdles, contributing to bundle or protocol adaptation, using performace data to guide practice change, and leading or participating in tests of change within multidiciplinary teams, rather than initial willingness to participate (“buy-in”).

Our context includes state Perinatal Quality Collaboratives (PQCs) and the Alliance for Innovation on Maternal Health (AIM/AIM-CCI). PQCs are state or multistate networks of multidisciplinary teams that implement continuous QI to improve maternal and infant outcomes, increasingly with an explicit equity focus [12, 13]. AIM develops and disseminates maternal safety bundles and implementation support that PQCs and participating sites adapt locally [7, 14, 15]. These structured programs provide external accountability, shared measurement, and technical support but still depend on sustained physician engagement at the site level to translate guidance into routine practice.

We used the Tailored Implementation for Chronic Diseases (TICD) framework as a theoretical lens to examine multilevel determinants of sustained physician engagment. TICD has been widely applied to identify barriers and facilitators to practice change across individual, organizational, and system levels. However, its application to sustained clinician engagement, particularly in obstetric quality improvement contexts, remains limited. Applying TICD in this study allowed us to systematically examine how determinants interact over time to support or constrain ongoing physician participation, while also identifying obstetric-specific factors that may extend existing implementation determinant frameworks [16, 17].

## Methods

### Study design

The study examined Obstetricians and Gynecologists’ (OBGYN) involvement in quality improvement projects. Using the TICD framework, this study employed semi-structured interviews to systematically categorize and analyze the determinants impacting physician participation in quality improvement initiatives. The TICD framework, which covers professionals, organizational structures, and healthcare systems, was selected for its comprehensive approach to addressing determinants impacting healthcare change implementation [16, 17]. Reporting follows the Consolidated Criteria for Reporting Qualitative Research (COREQ) [18], and study design/analysis were guided by the Tailored Implementation for Chronic Diseases (TICD) framework [17].

### Setting and participants

24 OBGYNs, with a minimum of one year's engagement in quality improvement projects and interdisciplinary team experience, were eligible to participate in this study. Participants were identified in collaboration with State Perinatal Quality Collaboratives (PQCs) and the Alliance for Innovation on Maternal Health (AIM/AIM-CCI) using distribution lists and partner referrals. These initiatives emphasize implementation of maternal safety bundles (e.g., obstetric hemorrhage, hypertensive disorders, sepsis, equity), simulation-based training, data-driven PDSA cycles, and multidisciplinary protocols across inpatient and outpatient settings [7, 14, 15]. Eligible physicians had ≥ 1 year of involvement in obstetric QI and participated in multidisciplinary QI activities in inpatient, outpatient, or mixed settings. We used purposive sampling to vary years in practice, geography, practice model (academic, community, safety-net), and clinical setting (labor and delivery, outpatient, or both). We targeted ~ 20–25 interviews anticipating thematic saturation; saturation was reached by interview 22 and confirmed with two additional interviews (final *N* = 24).

### Data collection

Interviews were conducted from November 2021 to August 2022. Remote interviews were conducted via Zoom. The interview guide was designed according to the TICD model, focusing on physician motivation, obstacles to involvement, leadership backing, resource sufficiency, and team collaboration in quality improvement projects. Open-ended questions were used during interviews, lasting between 30 and 60 min, for participants to share their experiences and viewpoints on QI project facilitators and barriers, which were transcribed verbatim for analysis. Participant data was anonymized to ensure confidentiality. The semi-structured interview guide, developed using TICD domains, is provided in Additional file 1.

### Researcher position and reflexivity

All interviews were conducted by the first author (SS), a male researcher with experience in maternal health policy and QI implementation, who had no prior relationships with participants. To mitigate positionality effects, we used neutral prompts, kept reflexive memos, engaged two coders, and held consensus meetings; transcript review (member checking) was offered to participants**.**

### Data analysis

We developed an initial deductive codebook from TICD constructs and iteratively added inductive codes emerging from early transcripts. Two researchers independently coded all transcripts using MAXQDA (VERBI, 2022) and met bi-weekly to resolve differences by consensus, maintaining an audit trail of codebook revisions and analytic decisions. Consistent with reflexive thematic analysis [19], we emphasized negotiated agreement rather than calculating inter-rater statistics. Themes are presented using TICD domain headings. A detailed construct-to-theme mapping with exemplar quotation IDs is provided in Additional file 2. Rigor was supported by dual coding, an audit trail, reflexive memoing, triangulation across diverse practice settings, and offered member checking.

### Ethical considerations

In November 2021, the Biomedical Research Alliance of New York (BRANY) IRB granted exempt status (Protocol # 21–121–938) for the study's review and approval. All participants provided informed consent prior to interview. Data were anonymized and securely stored to maintain confidentiality. Participants were informed they could decide to terminate their participation at any time. The participants were offered the opportunity to review the accuracy of the transcript during member checking [20]. The diverse clinical perspectives among participants were integrated to accomplish triangulation and better understand physician's perspectives [21]. We ensured reflexivity to mitigate biases during data collection and analysis. The analysis process ensured transparency through the maintenance of an audit trail that recorded decision-making.

## Results

In this study, physician engagement was defined as sustained participation in quality improvement (QI) activities over time, including attendance at QI meetings and huddles, contribution to bundle or protocol adaptation, use of performance data to guide practice change, and participation in tests of change within multidisciplinary teams. Using the Tailored Implementation for Chronic Diseases (TICD) framework, we identified facilitators and barriers influencing physicians’ continued engagement once involved in obstetric QI initiatives. Table 1 summarizes participant characteristics, and Table 2 presents key determinants of sustained physician engagement organized by TICD domain. Detailed construct-to-theme mappings with exemplar quotation identifiers are provided in Additional file 2.

**Table 1:** Participant characteristics

Characteristic	Value
Sample size (N)	24
Gender	Male 10 (42%)/Female 14 (58%)
Age Group	Under 55:13 (54%) Above 55: 11(46%)
Years in practice	0–5: 0/6–10: 5(21%)/11–15: 6(25%)/16–20: 5(21%)/> 20 years: 8(33%)
Years involved in QI	0–2: 0/3–5: 7(29%)/6–10: 8(33%)/> 10 years: 9 (38%)
Practice model	Academic: 21 (88%)/Community/Health system: 2 (8%)/Other: 1 (4%)
Subspecialty	MFM: 11 (46%); General OB-GYN: 11 (46%); Urogynecology: 1 (4%); Other: 1 (4%)
*Region*	Northeast (New England + Middle Atlantic): 8 (33%) Midwest (East + West North Central): 4 (17%) South (South Atlantic + East + West South Central): 7 (29%) West (Mountain + Pacific): 5 (21%)

**Table 2:** Determinants of Sustained Physician Engagement in Obstetric Quality Improvement, Organized by TICD Domain

TICD Domain	Theme	Role in Engagement	How the Determinant Influenced Sustained Physician Engagement
**Guideline Factors**	Flexible, evidence-based guidelines	Facilitator/Mixed	Adaptable guidelines allowed physicians to exercise clinical judgment while maintaining standardization, supporting continued participation; perceived rigidity or medico-legal concerns reduced willingness to remain engaged
	Co-production and ownership of protocols	Facilitator	Involvement in guideline and bundle development increased physicians’ sense of ownership and accountability, strengthening long-term commitment to QI activities
**Individual Health Professional Factors**	Patient-centered motivation	Facilitator	Seeing tangible improvements in maternal outcomes reinforced physicians’ intrinsic motivation to remain engaged over time
	Longitudinal QI knowledge and skills	Facilitator	Ongoing education in QI methods increased confidence and capacity to participate; lack of training constrained sustained involvement
	Burnout and competing clinical demands	Barrier	High clinical workload and emotional exhaustion limited physicians’ ability to consistently engage in QI activities
**Patient Factors**	Equity and disparity reduction	Facilitator	Awareness of persistent racial and socioeconomic disparities in maternal outcomes strengthened physicians’ moral commitment to sustained QI engagement
	Patient needs and care context	Facilitator/Mixed	Addressing patient barriers (e.g., access to postpartum care, home BP monitoring) motivated engagement but increased coordination burden affected sustainability
**Professional Interactions**	Multidisciplinary collaboration	Facilitator	Team-based collaboration, particularly nurse–physician partnerships, supported shared responsibility and sustained engagement
	Psychological safety and communication	Facilitator	Environments that encouraged open dialogue and learning enabled physicians to remain engaged despite implementation challenges
**Incentives and Resources**	Protected time and staffing	Facilitator/Barrier	Availability of protected time enabled participation; lack of time and staffing was a persistent barrier to sustained engagement
	Data infrastructure and audit-and-feedback	Facilitator	Access to timely, actionable data reinforced physicians’ perception of QI value and supported continued participation
	Recognition and career advancement	Facilitator	Formal recognition or inclusion of QI in promotion criteria legitimized engagement and supported sustained involvement
**Capacity for Organizational Change**	Participatory leadership	Facilitator	Leaders who co-set goals, aligned priorities, and secured resources created conditions for sustained physician engagement
	Organizational culture and strategic alignment	Facilitator/Barrier	Embedding QI into routine operations supported engagement; short-term or project-based approaches undermined sustainability
**Social, Political, and Legal Factors**	External accountability and mandates	Mixed	Collaborative requirements and policy expectations reinforced engagement, but misalignment with local workflows constrained participation
**Inductive, OB-Specific Themes**	L&D workflow intensity	Barrier	Unpredictable labor and delivery volume and on-call schedules directly limited physicians’ ability to attend meetings and lead tests of change
	Care continuity across prenatal–postpartum settings	Barrier	Transitions across care settings increased coordination demands, affecting physicians’ capacity to remain engaged over time
	Equity-driven professional identity	Facilitator	Commitment to addressing maternal health inequities uniquely motivated sustained engagement in obstetric QI initiatives

Percentages are calculated out of non-missing values; where applicable, ‘Unknown/Not reported’ is provided. Category counts sum to *N* = 24.

Themes are presented using TICD domains: Guideline Factors; Individual Health Professional Factors; Patient Factors; Professional Interactions; Incentives and Resources; Capacity for Organizational Change; and Social, Political, and Legal Factors.

### Guideline factors

Participants described guideline characteristics as influencing their sustained engagement in obstetric QI initiatives. Flexible, evidence-based guidelines supported continued participation by allowing physicians to apply clinical judgment while maintaining standardized care processes. In contrast, perceived rigidity of protocols and medico-legal concerns when deviating from guidelines constrained physicians’ willingness to remain engaged over time (Exemplar quotes: G1–G4, Additional file 2).

Participants also reported that involvement in bundle or protocol development strengthened their sense of ownership and accountability, which supported ongoing engagement. When protocols were perceived as externally imposed without opportunity for local adaptation, participants described lower motivation to remain actively involved in QI activities.

### Individual health professional factors

Individual-level determinants were described as central to sustained physician engagement (Table 2). Participants consistently described patient-centered motivation, including observing improvements in maternal outcomes, as a primary driver of continued involvement (Exemplar quotes: I1–I3). Confidence in QI methods varied across participants, and those with longitudinal exposure to QI training described greater capacity to remain engaged.

Participants also described competing clinical demands and burnout as constraining their ability to consistently participate in QI activities. High clinical workload and limited scheduling flexibility were described as limiting opportunities for sustained engagement despite intrinsic motivation.

### Patient factors

Participants described patient needs and care context as influencing sustained engagement by shaping implementation priorities and perceived relevance of QI initiatives (Table 2). Participants emphasized equity considerations, including persistent racial and socioeconomic disparities in maternal outcomes, as reinforcing their commitment to continued QI participation (Exemplar quotes: P1–P2).

Participants also described addressing patient-level barriers, such as access to postpartum follow-up care and home blood pressure monitoring, as increasing coordination demands. While these needs reinforced motivation to remain engaged, they also introduced logistical complexity that influenced physicians’ capacity to sustain involvement over time.

### Professional interactions

Participants described multidisciplinary collaboration as supporting sustained physician engagement (Table 2). Collaboration with nurses, midwives, administrators, and other professionals was described as enabling shared responsibility for QI work and supporting continued participation (Exemplar quotes: PR1–PR3).

Participants also described psychological safety and open communication as enabling continued engagement by allowing teams to address implementation challenges and adapt interventions over time. In contrast, siloed inpatient–outpatient workflows and staff turnover were described as disrupting continuity and constraining ongoing involvement in QI activities.

### Incentives and resources

Participants described availability of resources as influencing sustained engagement in obstetric QI initiatives (Table 2). Protected time, adequate staffing, and access to reliable data infrastructure were described as facilitating continued participation by supporting QI as part of routine clinical responsibilities (Exemplar quotes: IR1–IR3).

Participants also described time pressure, administrative burden, and competing institutional priorities as persistent barriers to sustained engagement. Formal recognition of QI participation within career advancement structures was described as supporting continued involvement.

### Capacity for organizational change

Participants described organizational leadership and culture as shaping conditions for sustained physician engagement (Table 2). Participatory leadership practices, including co-setting goals, aligning QI initiatives with institutional priorities, and securing resources, were described as supporting continued engagement (Exemplar quotes: OC1–OC3).

Participants also described short-term or project-based approaches to QI as less supportive of sustained engagement. Embedding QI into routine organizational processes was described as supporting continued participation over time.

### Social, political, and legal factors

Participants described external accountability mechanisms, including collaborative reporting requirements, payer expectations, and medico-legal context, as shaping implementation priorities and physician comfort with adaptation (Table 2) (Exemplar quotes: SPL1–SPL2).

Participants described policy engagement and advocacy as relevant to QI priorities but often beyond available time and capacity for most clinicians.

### Inductive, OB-specific themes

Participants described obstetric-specific determinants of sustained engagement that were not fully captured within TICD domains (Table 2). Labor and delivery workflow intensity, including unpredictable clinical volume and on-call schedules, was described as influencing attendance at QI meetings and ability to lead tests of change (Exemplar quotes: OB1–OB2).

Participants also described equity-driven professional identity as reinforcing continued engagement in obstetric QI initiatives. These themes are further described in Additional file 2.

## Discussion

Using the Tailored Implementation for Chronic Diseases (TICD) framework, this study identified multilevel determinants influencing sustained physician engagement in obstetric quality improvement (QI) initiatives among physicians already participating in structured collaborative programs. Participants described participatory leadership, multidisciplinary collaboration, patient-centered motivation, and adaptable guideline implementation as key facilitators of continued engagement. Persistent barriers included time constraints, administrative burden, competing clinical demands, and variability in data and infrastructure support. These findings align with established implementation determinant domains while also highlighting obstetric-specific contextual factors that influence sustained engagement [16, 17].

This study contributes to implementation science by characterizing sustained physician engagement as a longitudinal implementation determinant rather than an early implementation behavior. While prior implementation literature has primarily emphasized determinants of initial adoption and early participation, these findings demonstrate that sustained engagement is shaped by dynamic interactions across individual, organizational, and system-level determinants over time [8, 11, 22, 23]. By applying TICD in a population of physicians already participating in QI initiatives, this study extends determinant frameworks to examine factors influencing ongoing participation rather than initial implementation decisions [16, 17]. Additionally, the identification of obstetric-specific determinants, including workflow variability and equity-driven professional motivation, suggests that implementation determinant frameworks may require contextual adaptation within specialty clinical environments such as maternity care [16, 17].

### Guideline factors

Participants described guideline characteristics as influencing sustained engagement through their impact on clinical autonomy, perceived medico-legal risk, and feasibility of implementation in real-world obstetric settings [24, 25]. Flexible, evidence-based guidelines supported continued participation by allowing physicians to apply clinical judgment while maintaining standardized care processes [24]. In contrast, perceived rigidity of protocols and medico-legal concerns when deviating from guidelines constrained physicians’ willingness to remain engaged over time [26]. These findings align with implementation literature demonstrating that perceived intervention adaptability influences clinician participation and sustained implementation [9, 10].

Participants also described involvement in bundle or protocol development as strengthening ownership and accountability, supporting ongoing engagement. When protocols were perceived as externally imposed without opportunity for local adaptation, participants described lower motivation to remain actively involved in QI activities.

### Individual health professional factors

Individual-level determinants were described as central to sustained physician engagement. Participants consistently described patient-centered motivation, including observing improvements in maternal outcomes, as a primary driver of continued involvement. Confidence in QI methods varied across participants, and those with longitudinal exposure to QI training described greater capacity to remain engaged [27–29].

Participants also described competing clinical demands and burnout as constraining their ability to consistently participate in QI activities. High clinical workload and limited scheduling flexibility were described as limiting opportunities for sustained engagement despite intrinsic motivation. These findings align with prior literature demonstrating that clinician workload, competing priorities, and professional identity influence sustained participation in implementation and QI initiatives [10, 27, 29].

### Patient factors

Participants described patient needs and care context as influencing sustained engagement by shaping implementation priorities and perceived relevance of QI initiatives. Participants emphasized equity considerations, including persistent racial and socioeconomic disparities in maternal outcomes, as reinforcing their commitment to continued QI participation [2, 7, 30, 31].

Participants also described addressing patient-level barriers, such as access to postpartum follow-up care and home blood pressure monitoring, as increasing coordination demands. While these needs reinforced motivation to remain engaged, they also introduced logistical complexity that influenced physicians’ capacity to sustain involvement over time.

### Professional interactions

Participants described multidisciplinary collaboration as supporting sustained physician engagement [9, 32]. Collaboration with nurses, midwives, administrators, and other professionals was described as enabling shared responsibility for QI work and supporting continued participation [33]. Participants emphasized that sustained implementation in obstetric settings occurs within multidisciplinary team environments, particularly through nurse–physician collaboration, rather than through physician leadership alone [32, 34].

Findings also suggest that leadership and physician engagement represent distinct but related implementation constructs. Leadership functioned as an enabling determinant influencing resource allocation, institutional priorities, and organizational culture, whereas physician engagement reflected ongoing behavioral and relational participation in implementation activities [35, 36].

### Incentives and resources

Participants described availability of resources as influencing sustained engagement in obstetric QI initiatives. Protected time, adequate staffing, and access to reliable data infrastructure were described as facilitating continued participation by supporting QI as part of routine clinical responsibilities [9, 17, 37]. Participants also described time pressure, administrative burden, and competing institutional priorities as persistent barriers to sustained engagement. Formal recognition of QI participation within career advancement structures was described as supporting continued involvement.

### Capacity for organizational change

Participants described organizational leadership and culture as shaping conditions for sustained physician engagement. Participatory leadership practices, including co-setting goals, aligning QI initiatives with institutional priorities, and securing resources, were described as supporting continued engagement [16, 17]. Participants also described short-term or project-based approaches to QI as less supportive of sustained engagement. Embedding QI into routine organizational processes was described as supporting continued participation over time. These findings align with implementation research demonstrating that organizational readiness and leadership engagement influence implementation sustainability [10, 38].

### Social, political, and legal factors

Participants described external accountability mechanisms, including collaborative reporting requirements, payer expectations, and medico-legal context, as shaping implementation priorities and physician comfort with adaptation [17, 39]. Participants also percieved policy engagement and advocacy as relevant to QI priorities but often beyond available time and capacity for most clinicians [22, 40].

### Obstetric-specific considerations

Participants described obstetric-specific determinants of sustained engagement that were not fully captured within TICD domains. Labor and delivery workflow intensity, including unpredictable clinical volume and on-call schedules, was described as influencing attendance at QI meetings and ability to lead tests of change [26, 41, 42]. Participants also described continuity challenges across prenatal, intrapartum, and postpartum care, particularly for initiatives extending beyond inpatient settings. These transitions increased coordination demands and influenced physicians’ capacity to remain engaged without adequate infrastructure support [30, 43–45]. Equity-driven professional motivation emerged as a distinctive facilitator of sustained engagement, with participants describing commitment to addressing maternal health disparities as reinforcing continued involvement in QI activities [42].

### Implications

Participants’ perspectives suggest several implementation-relevant strategies to support sustained physician engagement. First, embedding QI within clinical roles through protected time, clear expectations, and alignment with recognition and advancement criteria may improve acceptability, feasibility, and sustainability of physician participation. Second, providing longitudinal QI training, including methods, equity-focused adaptation, and routine performance data use from residency through practice, may support clinician self-efficacy and sustained implementation fidelity over time. Third, participatory leadership approaches that involve physicians in co-setting goals and tailoring bundles may support adoption and penetration by ensuring implementation guidance is both standardized and adaptable to local clinical context.

### Strengths and Limitations

The sample included physicians already engaged in QI, which likely overrepresents champions and underestimates barriers among non-participants. Perspectives from other stakeholders (e.g., midwives, nurses, patients) were not included, limiting transferability to fully multidisciplinary settings. Using TICD provided structure but may have constrained inductive insights; we mitigated this by labeling inductive themes and providing a construct mapping (Additional file 2). Findings are self-reported; triangulation with observational or site-level performance data was beyond scope. Future research is needed to prospectively test the three strategies above and evaluate implementation outcomes (e.g., acceptability, adoption, feasibility, fidelity, penetration, sustainability) and equity impacts using established outcome taxonomies.

## Conclusion

Physicians already engaged in obstetric QI described participatory leadership, multidisciplinary collaboration, and patient-centered motivation as key facilitators of sustained engagement, while limited time, administrative burden, and variable data support were the most persistent barriers. Grounded in TICD domains, these findings highlight three implementation-relevant priorities: (1) embedding QI within clinical roles and protected time; (2) providing longitudinal QI training in methods, routine data use, and equity-focused adaptation; and (3) using participatory leadership approaches to co-adapt bundles so guidance remains standardized while adaptable to local clinical context. Implementation strategies aligned with these priorities may improve acceptability, feasibility, and sustainability of physician participation and support long-term engagement within PQC/AIM implementation environments, with potential downstream impact on maternal health disparities. 

## Supplementary Information

Additional file 1.

Additional file 2.

## Data Availability

Interview data and study materials have not been approved to be used for other studies based on informed consent obtained from participants.
