# Supplementary material for: Determinants of sustained physician engagement in obstetric QI: a TICD-guided qualitative study
Source: Implement Sci Commun. 2026 Mar 14;7:80. doi: 10.1186/s43058-026-00898-y (PMC13104307; doi:10.1186/s43058-026-00898-y)
Supplement: Supplementary file 1 — Additional file 1. [file 43058_2026_898_MOESM1_ESM.docx]

**Interview Guide**

Participant No: ______

Gender: _____

Age_______

Specialty: ________

Years of participating in the state AIM collaborative: ________

Approximate number of published peer-reviewed papers in the literature on maternal child health (MCH) subject area:_________

**INTRODUCTION**

Thank you for accepting my invitation to be interviewed in your capacity as a scholar and subject matter expert or participant within the AIM/PQC collaborative. The lack of practitioner-based knowledge on factors that aid in effective implementation of quality improvement by multidisciplinary teams. The purpose of this interview and study to understand the factors that directly or indirectly impact providers' approach to participating and engaging in the interdisciplinary quality improvement initiative.

In this study, we consider multidisciplinary quality improvement as a system "systematic, formal approach to the analysis of practice performance and efforts to improve performance" participants a multidisciplinary better to understand providers' diverse perspective and experience. With your permission, I’d like to record this interview. The recording would be for learning purposes only and would be destroyed at the end of my research. Do you have any questions for me, before we begin?

**BACKGROUND**

- Let’s start with having you describe your role in the Collaborative.
- How did you get involved with the Collaborative?
- How long have you been involved?
- Has your role changed over time? In what ways?
- What specific aspects of the initiative attracted you to this work?

**ATTITUDES & BEHAVIORS**

- How would you describe multidisciplinary to a work colleague, i.e., what does
- multidisciplinary mean to you?
- What are your perceptions on practical approaches to multidisciplinary collaborative
- initiatives like AIM or PQC?
- What is your approach to getting providers in your specialty to be more engaged at the community and hospital level in Quality Improvement Initiatives?

**CRITICAL ISSUES**

- In your experience, what are the barriers to the effective implementation of evidence
- based recommendations?
- What do you think are the main drivers of maternal mortality and morbidity in your
- community?
  - Are there people or organizations addressing these issues?
  - Are these issues getting better or worse in the community?

**FUTURE DIRECTION**

- Can you reflect on what attracted you to this work in the first place?
- What keeps you motivated to continue participating?
- What areas do you think collaboratives should explore for improvements?
- How can new collaboratives improve engagement with providers in your specialty?
- How can multidisciplinary collaboratives improve engagement with providers?

**CLOSING**

Thank you for assisting me with this research study. I will contact you via email once the transcript of the interview is finalized. I will provide a summary of the discussion, and I would like you to review the summary as confirmation that I have captured the essence of what you have shared with me. If any discrepancies are found, I will correct the interpretations. Do you have any questions? Please contact me if you have any questions.
