# Supplementary material for: Determinants of sustained physician engagement in obstetric QI: a TICD-guided qualitative study
Source: Implement Sci Commun. 2026 Mar 14;7:80. doi: 10.1186/s43058-026-00898-y (PMC13104307; doi:10.1186/s43058-026-00898-y)
Supplement: Supplementary file 2 — Additional file 2. [file 43058_2026_898_MOESM2_ESM.docx]

**Consolidated Criteria for Reporting Qualitative Research (COREQ) Checklist**
*Based on the study: "Understanding Physicians’ Perspectives on the Effectiveness of Multidisciplinary Quality Improvement (QI) Strategies to Address Disparities in Maternal Health"*

**Domain 1: Research Team and Reflexivity**

**Personal Characteristics**

1. **Interviewer/facilitator:** The interviews were conducted by Saanie Sulley, a researcher experienced in quantitative and qualitative methodologies and maternal health policy.
2. **Credentials:** The lead interviewer holds a dual doctorate of Medicine (MD) and philosophy (PhD) with expertise in informatics and maternal health quality improvement initiatives.
3. **Occupation:** The interviewer is a researcher affiliated with National Healthy Start Association and a Visiting Scholar with the American Board of Medical Specialties during the study.
4. **Gender:** The interviewer was a male.
5. **Experience and training:** The interviewer has extensive experience conducting quantitative and qualitative research, specifically in health services research and QI implementation studies.

**Relationship with Participants**

1. **Was a relationship established prior to study commencement?** No prior relationship existed between the researcher and participants.
2. **Participant knowledge of the interviewer:** Participants were informed that the interviewer was conducting a study on physician engagement in QI.
3. **Interviewer characteristics influence on research:** Steps were taken to minimize bias, including reflexivity and using a structured interview guide.

**Domain 2: Study Design**

**Theoretical Framework**

1. **Methodological orientation and theory:** The study used the **Tailored Implementation for Chronic Diseases (TICD) framework** to analyze physician engagement in QI initiatives.

**Participant Selection**

1. **Sampling method:** Purposive sampling was used to recruit obstetricians and gynecologists engaged in QI initiatives.
2. **Method of approach:** Physicians were invited via email from professional networks and QI program affiliations.
3. **Sample size:** 24 physicians participated.
4. **Non-participation:** Some invited participants declined due to scheduling conflicts.

**Setting**

1. **Setting of data collection:** Interviews were conducted remotely via Zoom between November 2021 and August 2022.
2. **Presence of non-participants:** Only the interviewer and the participant were present during each interview.
3. **Description of sample:** Participants included obstetricians and gynecologists involved in QI initiatives across different healthcare settings.

**Data Collection**

1. **Interview guide:** A semi-structured interview guide was developed based on the TICD framework.
2. **Repeat interviews:** No repeat interviews were conducted.
3. **Audio/visual recording:** Interviews were recorded and transcribed verbatim.
4. **Field notes:** Researchers took field notes during interviews.
5. **Duration:** Interviews lasted between 30 and 60 minutes.
6. **Data saturation:** Data saturation was reached when no new themes emerged.
7. **Transcripts returned:** Participants were given the opportunity to review and validate transcripts (member checking).

**Domain 3: Analysis and Findings**

**Data Analysis**

1. **Number of data coders:** Two independent researchers coded the transcripts.
2. **Coding process:** Thematic analysis was conducted using **MAXQDA software** based on the TICD framework.
3. **Derivation of themes:** Themes were derived both inductively from participant narratives and deductively from the TICD framework.
4. **Software:** MAXQDA was used for data organization and analysis.
5. **Participant checking:** Member checking was conducted to ensure accuracy of interpretations.

**Reporting**

1. **Quotations presented:** Relevant participant quotes were included to illustrate key themes.
2. **Data and findings consistency:** The findings align with established implementation science literature and physician engagement studies.
3. **Clarity of major themes:** Themes were clearly defined and supported by participant quotes.
4. **Clarity of minor themes:** Sub-themes were identified and contextualized within the broader analysis.

**Checklist Completion**

- This COREQ checklist is submitted as an **Additional File** with the manuscript, as required by *Implementation Science*.
- The study adhered to best practices for qualitative research to ensure transparency and rigor in reporting.
